# Toward Unifying Evolutionary Ecology and Genomics to Understand Positive Plant–Plant Interactions Within Wild Species

**DOI:** 10.3389/fpls.2021.683373

**Published:** 2021-07-09

**Authors:** Harihar Jaishree Subrahmaniam, Dominique Roby, Fabrice Roux

**Affiliations:** LIPME, INRAE, CNRS, Université de Toulouse, Castanet-Tolosan, France

**Keywords:** intraspecific variation, genotype-by-genotype interactions, kin cooperation, diversity-productivity relationship, overyielding

## Abstract

In a local environment, plant networks include interactions among individuals of different species and among genotypes of the same species. While interspecific interactions are recognized as main drivers of plant community patterns, intraspecific interactions have recently gained attention in explaining plant community dynamics. However, an overview of intraspecific genotype-by-genotype interaction patterns within wild plant species is still missing. From the literature, we identified 91 experiments that were mainly designed to investigate the presence of positive interactions based on two contrasting hypotheses. Kin selection theory predicts partisan help given to a genealogical relative. The rationale behind this hypothesis relies on kin/non-kin recognition, with the positive outcome of kin cooperation substantiating it. On the other hand, the elbow-room hypothesis supports intraspecific niche partitioning leading to positive outcome when genetically distant genotypes interact. Positive diversity-productivity relationship rationalizes this hypothesis, notably with the outcome of overyielding. We found that both these hypotheses have been highly supported in experimental studies despite their opposite predictions between the extent of genetic relatedness among neighbors and the level of positive interactions. Interestingly, we identified a highly significant effect of breeding system, with a high proportion of selfing species associated with the presence of kin cooperation. Nonetheless, we identified several shortcomings regardless of the species considered, such as the lack of a reliable estimate of genetic relatedness among genotypes and ecological characterization of the natural habitats from which genotypes were collected, thereby impeding the identification of selective drivers of positive interactions. We therefore propose a framework combining evolutionary ecology and genomics to establish the eco-genomic landscape of positive GxG interactions in wild plant species.

## Introduction

During the course of its life cycle, a plant can interact directly or indirectly – consecutively and/or concurrently – with multiple neighboring plants. Plant social networks include interactions among individuals of different species (i.e., interspecific interactions) and among genotypes of the same species (i.e., intraspecific interactions) in a local environment. Plant–plant interactions play an important role in regulating the diversity and structure of plant communities and ultimately ecosystem functioning, through their effects on resource availability and habitat structure (Brooker, 2006; Martorell and Freckleton, 2014). Studying the mechanisms underlying plant–plant interactions is therefore essential to understand the dynamics of plant communities, which may in turn help to predict the resilience of plant species to anthropogenic-related global changes (Subrahmaniam et al., 2018). For instance, ongoing climate warming has resulted in modifications of plant assemblages due to increase of plant biomass, reduced diversity (Baldwin et al., 2014) and shifts in the distribution areas of plant species (Gilman et al., 2010; Singer et al., 2013).

Plant–plant interactions can be divided into four main categories depending on the net benefit and cost associated with the interaction (Subrahmaniam et al., 2018). Firstly, competitive interactions (–/–) are those that come with a significant cost for both partners (benefit < 0, cost > 0 for both partners). Competition is characterized by reciprocal negative effects on plant growth or fitness caused by the presence of neighbors (Keddy, 2015). Since all plants share few basic requirements, limitations of resources such as the availability of nutrients, water or light could drive competition among plants (Turkington and Harper, 1979; Chaney and Baucom, 2014). Second, asymmetric interactions (–/+) yield benefit to one of the partners, at the cost of the other interactor (benefit < 0 and cost > 0 for the helper; benefit > 0 and cost < 0 for the receiver). Parasitic plants are the prime example of this kind of behavior. In addition, plants releasing allelochemicals, which negatively influence the physiology of their neighbors, can be grouped under this category. Third, commensal interactions (0/+) are those that are beneficial for one of the partners, but there is no cost associated with providing such aid (benefit = 0 and cost = 0 for helper; benefit > 0 and cost = 0 for the receiver of the help). Many examples of such interactions exist at the interspecific level, like nurse plant effects in deserts or climbing plants that use the stems of other plants to avoid shade (Padilla and Pugnaire, 2006; Gianoli, 2015). Lastly, individuals can also reciprocally benefit (+/+) from being associated with a partner (such that the net benefit > 0 for each plant partner). One of the first examples demonstrating this mutually beneficial relationship came from a leguminous shrub, *Retama sphaerocarpa* and its understory species *Marrubium vulgare* (Pugnaire et al., 1996b). Increasing resource availability leading to facilitative benefits of being underneath the shrub, was reciprocated by improved water relationships due to the understory species in this study.

Estimating the relative importance of these broad categories in explaining patterns of plant communities is still under debate and mainly focused on interactions at the interspecific level. Interspecific competitive interactions have been traditionally recognized as the major factors driving the structure (Goldberg and Barton, 1992), diversity (Chesson, 2000) and dynamics of plant communities (Tilman, 1985). However, the role of positive interactions among species (including both commensal interactions and reciprocal helping interactions) in regulating the composition of communities, has recently gained attention (Bertness and Callaway, 1994; Callaway, 1995; Brooker and Callaghan, 1998; Dormann and Brooker, 2002; Bruno et al., 2003; Kotowska et al., 2010; Wendling et al., 2017). In particular, positive interactions among species have been put forward to explain overyielding, a phenomenon which corresponds to the increase in productivity of species when grown in a mixture, as opposed to monoculture (Harper, 1977; Vandermeer, 1981; Loreau, 2004; Schmid et al., 2008). Also being increasingly recognized is the notion that studying Genotype-by-Genotype (GxG) interactions (also referred as intergenotypic interactions) at the intraspecific level might be a prerequisite for understanding eco-evolutionary patterns of plant communities (Hughes et al., 2008). Indeed, a huge number of genotypes of varying levels of genetic relatedness can co-exist within a local population, even in the case of highly selfing species. For instance, a recent study on *Arabidopsis thaliana* revealed that the genetic diversity observed within a local population can represent almost one-sixth of the genetic diversity at the worldwide scale (Frachon et al., 2017). Consequently, the patterns of interactions between different genotypes within one population are bound to vary as well.

Several meta-analyses have been carried out to understand patterns of GxG interactions at the interspecific level in herbaceous wild plant species (Maestre et al., 2005) as well as in trees (Piotto, 2008; Zhang et al., 2012). However, to our knowledge, an overview of GxG interaction patterns at the intraspecific level within wild plant species is still missing from literature. We therefore aimed to make a synthesis on such interactions. By surveying the literature, we quickly observed that most studies have been dedicated to testing the presence of positive interactions within different plant species. Moreover, studies on these positive GxG interactions have been based upon testing two main non-exclusive hypotheses with opposite relationships between the extent of genetic relatedness among neighbors and the level of positive interactions (File et al., 2012): kin/non-kin recognition or elbow-room. Rooted in evolutionary biology concepts, the first hypothesis is based on the kin selection theory advocating that individuals increase their inclusive fitness by modifying their behavior to help a relative, thereby leading to *kin cooperation* (Hamilton, 1964). On the other hand, rooted in ecological concepts, the elbow-room hypothesis assumes that intraspecific resource partitioning occurs and increases as the genetic distance between neighbors increases (Argyres and Schmitt, 1992). The resulting expected positive relationship between genotypic diversity and productivity corresponds to *overyielding* at the intraspecific level.

After highlighting the nature of the data of 78 identified articles and identifying the main caveats before extracting trends from these data, we tested whether kin cooperation and overyielding observed within our sample dataset was dependent upon specific features of a species (e.g., breeding system, mode of seed dispersal, life cycle etc.). Finally, we introduce several avenues that deserve to be explored to obtain a thorough picture of GxG interaction patterns within wild species. We particularly stress the need to integrate genomics and evolutionary ecology to fully understand the complexity of intraspecific positive genetic interactions in wild plant populations.

## Literature Survey and Hypotheses Tested

**FIGURE 1:**
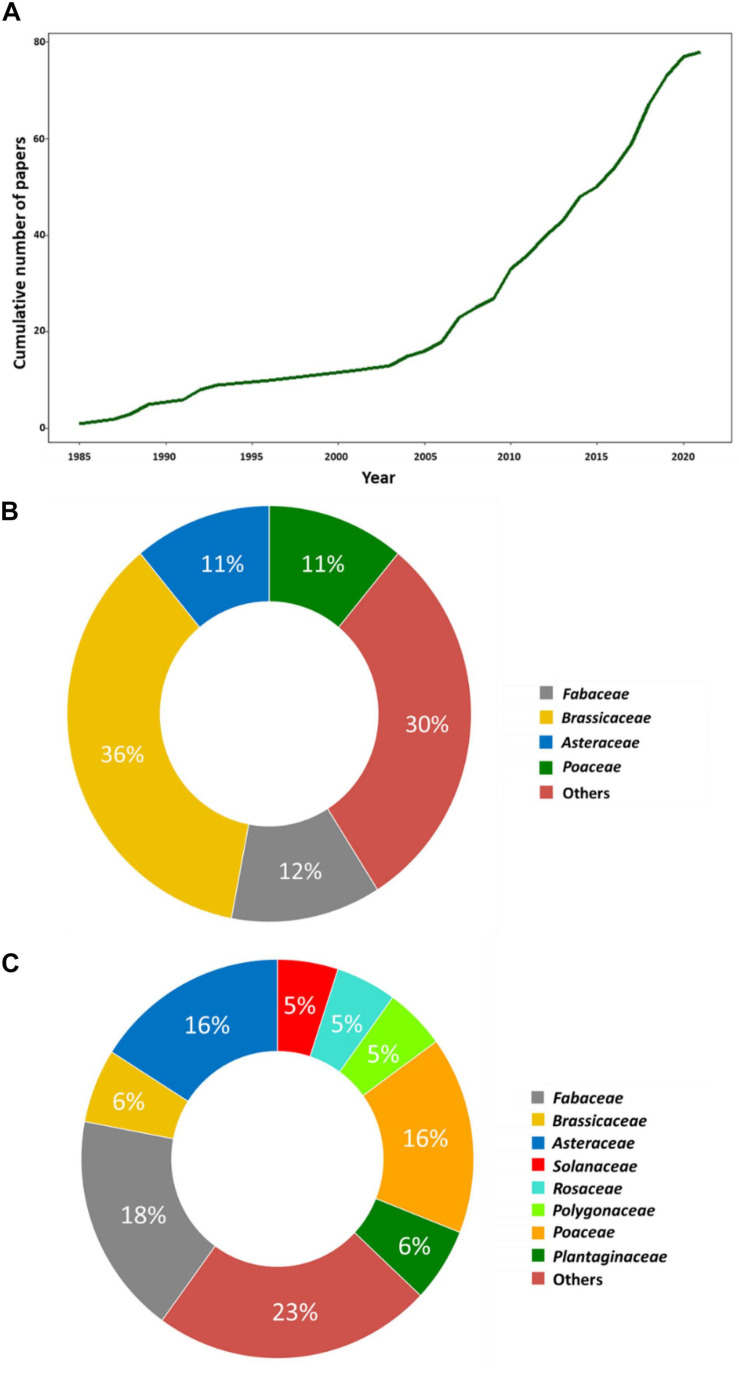
Surveying GxG interactions within herbaceous wild species. **(A)** Cumulative number of papers on GxG interactions in wild plant species from 1985 to 2021. **(B)** Doughnut plots describing the distribution of the major botanical families used in the study of intraspecific GxG interactions. **(C)** Doughnut plots describing the distribution of the major botanical families used in the study of intraspecific GxG interactions upon removal of *Arabidopsis thaliana*.

For this review, we only focused on studies looking at intraspecific interactions within wild populations of herbaceous species. We made this choice because the number of generations of wild herbaceous species is clearly lesser than the one of trees. Therefore, fitness proxies can be better estimated during their life cycle. Several keywords were used to gather these studies, i.e., ‘GxG interactions,’ ‘intraspecific interactions,’ ‘intraspecific variation,’ ‘intra/inter-population variation,’ and ‘group selection.’ The websites inspected included Google Scholar, Web of Science, Sci-hub, as well as the web application Researcher^1^. Although we tried to do a comprehensive analysis to include a maximum number of studies reporting intraspecific GxG interactions, the list is certainly not exhaustive, and some studies may have been overlooked. We gathered a list of 78 articles published in the last 36 years and including 91 experiments (Supplementary Table 1). Interestingly, we observed a sharp increase in cumulative number of experimental papers published over the years, notably in the last 15 years (Figure 1A). This illustrates the rising interest in examining intraspecific GxG interactions within wild plant species.

The list includes 47 species belonging to 19 botanical families (Figure 1B). The most commonly studied botanical families are the Brassicaceae (36%), Fabaceae (12%), Asteraceae (11%), and Poaceae (11%). However, there is a significant bias in the family Brassicaceae toward *A. thaliana* as it constitutes about ∼88% of the studies from this family. Upon removing this species, the relative proportion of botanical families studied is more consistent with the number of studied species within each family (Figure 1C and Supplementary Table 1).

**FIGURE 2:**
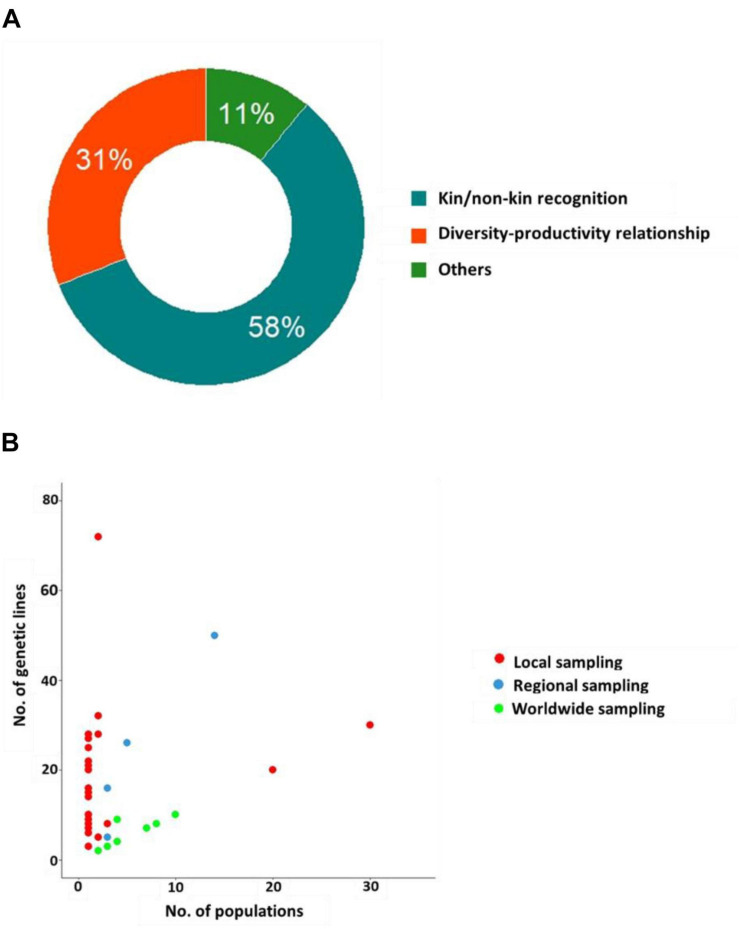
Classification of experimental studies on GxG interactions. **(A)** Doughnut plot describing the distribution of the papers according to the rationale of studying GxG interactions. **(B)** Relationship between the number of genetic lines and the number of populations according to three geographic scales. For better illustration, the two outliers with 60 genotypes each from a different population, are not incorporated in the figure (Fitzpatrick et al., 2019; Turner et al., 2020).

The reported experiments can be categorized upon testing either the ‘kin/non-kin recognition’ hypothesis (∼58% of the experiments) where the differential response of a genotype was tested in the presence/absence of a relative genotype (kin) *vs.* a stranger genotype in pairwise experiments, or the elbow-room (or ‘genotypic diversity-productivity relationship’) hypothesis (31% of the experiments) where fitness proxies were compared between monocultures using multiple kin individuals (compound intra-genotypic interactions) and mixtures of different genotypes (compound inter-genotypic interactions) (Figure 2A and Supplementary Table 1).

The remaining experiments (∼11%) were grouped under the category ‘Others’. These experiments aimed at (i) characterizing the genetic architecture underlying GxG interactions (Botto and Coluccio, 2007; Mutic and Wolf, 2007), (ii) studying the effects of GxG interactions on intra-individual traits such as genome size variation (Šmarda et al., 2010) and transcriptomic profiles (Bowsher et al., 2017), (iii) studying extended phenotypes such as root exudate profiles (Badri et al., 2012) and soil microbial communities (Burghardt et al., 2019; Fitzpatrick et al., 2019), (iv) testing the effect of adding a neighbor plant on genotype-by-environment interactions (i.e., GxExG instead of GxGxE) (Cahill et al., 2010), (v) examining local adaptation of genotypes (Linhart, 1988; Espeland and Rice, 2007), and (vi) investigating individual *vs.* group selection in wild plant populations (Goodnight, 1985; Donohue, 2003). For the latter, we need to stress that the existence of group selection is still controversial (Nowak, 2006; Nowak et al., 2010; Rousset and Lion, 2011; Kramer and Meunier, 2016) and it will not be addressed in this review.

## Highlights of the Nature of the Data and Identification of Caveats

### Life-History Traits

The list of 47 species is divided roughly equally between annuals (∼46%) and perennials (∼54%) (Supplementary Table 1) and is dominated by selfing species that make up about 44% of the dataset. The remaining species represent species with mixed breeding systems (∼27%), outcrossing species (∼19%) and clonal species (∼10%). Allochory (assisted seed dispersal) is predominant in this list as about ∼60% of the listed species exhibited this mode of seed dispersal, while only ∼40% of listed species have an autochorous (self) mode of seed dispersal (Supplementary Table 1).

### Nature and Extent of Genetic Variation

Around 86% of the experiments listed were based on genotypes collected in natural populations (Supplementary Table 1). With the main goal of dissecting the underlying genetic and molecular mechanisms of GxG interactions, the remaining experiments were based on (i) experimental populations such as F_2_ populations, families of Recombinant Inbred Lines (RILs) or Introgression lines (IL, ∼9%) (Goodnight, 1985; Griffing, 1989; Botto and Coluccio, 2007; Mutic and Wolf, 2007; Willis et al., 2010; Latzel et al., 2013; Wuest and Niklaus, 2018; Hauri et al., 2021), and (ii) mutant lines (∼5%) (Cahill et al., 2005; Crepy and Casal, 2015; Wagg et al., 2015; Zhang and Tielbörger, 2019; Supplementary Table 1). Unsurprisingly, given the great amount of genetic resources publicly available, all these experiments concern *A. thaliana*, with the exception of *Trifolium pratense* (Wagg et al., 2015) and *Solanum lycopersicum* (Hauri et al., 2021).

For experiments based on genotypes collected in natural populations, we observed a clear tradeoff between the number of genotypes used and the number of populations sampled (Figure 2B). The number of natural genetic lines used to evaluate GxG interactions was highly variable among experiments, ranging from 2 to 72 (mean ∼12) (Figure 2B). On average, these lines have been collected from about four populations (min = 1, max = 60) (Figure 2B). In wild plant populations, intraspecific neighbors share common space over generations, and this increases their probability for repeated interactions. Consequently, positive interactions are more likely to evolve between members of a single population rather than between members of different populations (Nowak, 2006). Accordingly, most experiments that do not employ *A. thaliana* are based on natural genetic lines from a single population (∼75%) or sampled at a regional scale (between 2 and 14 populations, ∼11%). On the other hand, an opposite trend is observed across the 29 experiments performed on *A. thaliana*. Almost 90% of the experiments on this species utilized genotypes coming from worldwide collections. The main hypothesis to explain this bias in using worldwide genotypes in *A. thaliana* might be related to its predominantly selfing breeding system, which initially suggested that most populations were monomorphic (Platt et al., 2010). Therefore, the large public collections of genotypes that are available for *A. thaliana* mostly correspond to one representative genotype per population. However, progressively, a substantial number of studies challenged this view by revealing extensive genetic diversity within populations (Le Corre, 2005; Jorgensen and Emerson, 2008; Bomblies et al., 2010; Platt et al., 2010; Kronholm et al., 2012; Brachi et al., 2013; Roux and Bergelson, 2016; Frachon et al., 2017, 2018, 2019; Fulgione et al., 2018), thereby giving an opportunity of studying more relevant GxG interactions in *A. thaliana* at the local scale.

Based on all the experiments listed in this survey, we nonetheless identified a major shortcoming of the plant material used to study GxG interactions, regardless of the identity of the species considered. As previously mentioned, testing both the kin/non-kin recognition and elbow-room hypotheses requires an estimation of the degree of genetic relatedness among interacting genotypes. Kin selection theory predicts partisan help given to close relatives. By contrast, according to the elbow-room hypothesis, genetically close relatives will compete for the same resources and increasing genetic distance between genotypes can translate into increasing niche partitioning. Testing for these contrasting predictions would require integrating information about the extent of genetic relatedness among interacting genotypes. However, this crucial information has been poorly considered in these experiments (but see Crutsinger et al., 2006, 2008), which may question the realism of having tested interactions between different genotypes in some studies, in particular when genotypes were originating from a single population (Supplementary Table 1).

### Relevant Growth Conditions?

Performing experiments in controlled and field conditions are complementary (Bergelson and Roux, 2010; Brachi et al., 2010). Experiments conducted under controlled conditions drastically reduce environmental noise, thereby allowing the establishment of a direct link between phenotypic observations and genotype performance under a given set of stable environmental conditions. On the other hand, in the field, plants are exposed to a greater but more ecologically realistic range of abiotic and biotic fluctuations than typically encountered in controlled conditions. Nonetheless, encompassing all these environmental fluctuations requires the field experiments to be repeated over several years.

In our survey, ∼82% of the experiments were conducted under controlled conditions (Supplementary Table 1). Out of these, ∼55 and ∼20% of the experiments were conducted in greenhouse conditions and growth chambers (including root chambers and growth tunnels), respectively. The remaining experiments (∼7%) were performed under *in vitro* conditions. On the other hand, few experiments (∼14%) had been conducted under field conditions, fewer in the native habitats (only two reported experiments, Supplementary Table 1). Finally, four experiments (∼4%) were conducted in both greenhouse and field conditions (Espeland and Rice, 2007; Andersson, 2014; Ehlers et al., 2016). The type of growth conditions used to study intraspecific GxG interactions is therefore strongly biased in favor of controlled conditions, notably when compared to other types of biotic interactions. For instance, in a recent review on Genome-Wide Association studies (GWAS) performed on plant – pathogen interactions, 60% of the studies were conducted in controlled conditions (greenhouse/growth chambers) and 40% under field conditions (Bartoli and Roux, 2017).

Of particular note, around 68% of the experiments in our survey tested the effect of a particular environmental factor on GxG interactions, either in controlled or field conditions (Supplementary Table 1). Around 55% of these experiments tested the effect of abiotic factors, including light quality, nutrient status, CO_2_ concentration and drought. On the other hand, ∼50% of these experiments tested the effect of biotic factors, which mainly encompassed density and soil conditioning by one or a mixture of genotypes, in particular to investigate the diversity-productivity relationship (Bukowski and Petermann, 2014; Semchenko et al., 2017; Bukowski et al., 2018). Unfortunately, the treatments applied may not have been ecologically relevant. Indeed, in our survey, only ∼19% of the experiments loosely described the ecology of the populations used in the experiments (Supplementary Table 1). At most, only a rough description of habitats from which the genotypes were collected, was given. Since interspecific positive plant–plant interactions have been postulated to evolve in natural settings including a certain level of abiotic and/or biotic stress (i.e., ‘stress gradient hypothesis’; Bertness and Callaway, 1994; Brooker and Callaghan, 1998; Bruno et al., 2003), this is of particular importance to finely describe the ecology of native habitats from which genotypes are collected. This may in turn allow testing the stress gradient hypothesis at the intraspecific level.

### A Large Diversity of Phenotyped Traits

All the traits measured across the 91 experiments are listed in Supplementary Table 2. To assess GxG interactions, an average of 3.5 traits per study have been directly measured on plants (min = 1, max = 10). We divided the list of traits into four broad categories, each relating to a distinct eco-function of the plant (i.e., root related traits, shoot related traits, life history traits and seed production related traits). While ∼34% of the experiments scored life history related traits (e.g., germination and flowering timing), ∼53 and ∼76% of the experiments measured root (e.g., root length and biomass) and shoot (e.g., plant height and dry biomass) related traits, respectively. Seed production related traits (e.g., number of fruits and number of seeds per fruit) were measured in ∼40% of the experiments. Interestingly, most experiments focused on collecting phenotypic information using either two (∼46%) or three (∼20%) categories. About 40% of the experiments looked at both root and shoot related phenotypes, while ∼29% of experiments focused on both shoot and life history related traits. Only four experiments (∼4%) focused on all four categories (Willson et al., 1987; Linhart, 1988; Masclaux et al., 2010).

Another interesting category of traits corresponds to extended phenotypes, which contains traits that were not directly phenotyped on the interacting individuals but rather looked at alteration of the immediate environment in GxG interactions. In our survey, ∼14% of studies examined such extended phenotypes, which mainly focused on either studying soil microbial communities (File et al., 2012; Wagg et al., 2015; Abdala-Roberts et al., 2017; Burghardt et al., 2019; Tomiolo et al., 2020), tritrophic interactions (Crutsinger et al., 2006, 2008; Kotowska et al., 2010; Karban et al., 2013; Abdala-Roberts et al., 2017; Hauri et al., 2021) or biochemical properties of plant secreted compounds (Badri et al., 2012; Kalske et al., 2019).

**FIGURE 3:**
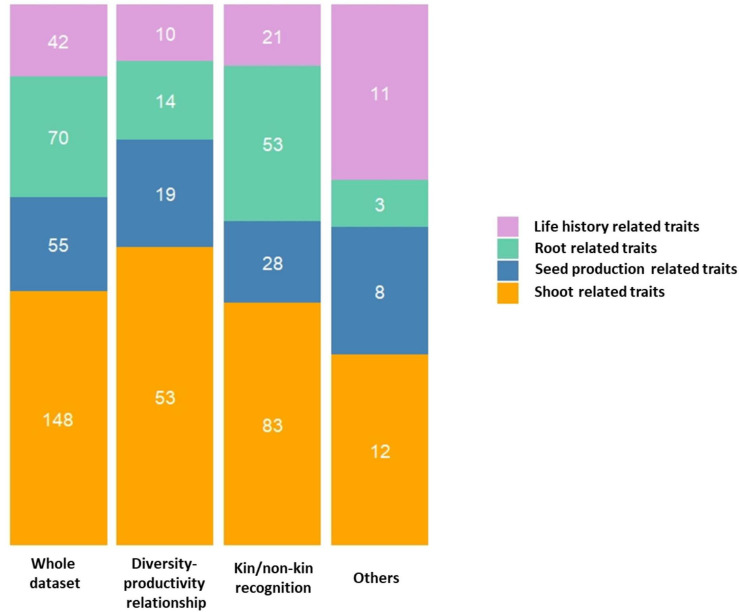
Stacked barplots illustrating the distribution of studies based on trait categories according to different rationales. The number of traits studied within each category are indicated within each stack.

When considering experiments testing the kin/non-kin recognition hypothesis, root related traits were phenotyped significantly more often than in experiments testing the diversity-productivity relationship (contingency chi-squared test, χ^2^ = 154.59, df = 3, *P* = 2.68 E-33, Figure 3). A potential explanation for this stems from the extensive literature on kin recognition studies, where many studies have demonstrated kin recognition by roots and subsequent nepotistic response across the plant kingdom (reviewed in Supplementary Tables 1, 2). On the other hand, experiments testing the diversity-productivity relationship examined more shoot and seed production related traits than experiments pertaining to kin/non-kin recognition (Figure 3). A potential explanation for this observation could be linked to the difficulties associated with measuring individual specific root response in experiments testing for the diversity-productivity relationship. Often, to measure specific root traits, the total root biomass of several genotypes present in a pot has been considered as a dependent measure, which obviously impedes the estimation of the relative contribution of each individual genotype in the pot. Therefore, aboveground traits could be an easier measure for quantifying productivity.

Plant neighbor interactions are often dynamic in nature, and can switch from competitive to positive interactions depending on (i) the environmental stress perceived (Bertness and Callaway, 1994), (ii) life stages of interacting species (Walker and Vitousek, 1991; Kellman and Kading, 1992; Chapin et al., 1994; Pugnaire et al., 1996a), or (iii) the physiological capacity of the interacting species (e.g., facilitation by improving plant water uptake under canopy shade or soil oxygenation; Callaway and King, 1996; Holmgren et al., 1997). In all the experiments listed in our survey, this potential switch of interactions at the intraspecific level has been overlooked. Testing for such complex dynamic GxG interactions would require phenotypic measurements of various plant compartments at multiple time points.

### Direct vs. Indirect GxG Interactions

We further categorized the experiments depending on whether the interactions tested were direct or indirect. We classified the interactions to be indirect when the experiment did not include placing at least two genotypes in a pot together or to allow any direct contact between them. In our survey, such indirect interactions have been addressed in ∼10% of the 91 experiments (Supplementary Table 1). Indirect interactions between genotypes may occur by two main processes. Firstly, soil conditioning can mediate indirect interactions between genotypes. There is mounting evidence suggesting the role of root exudates in identity recognition of neighbors. In our survey, two studies confirmed kin recognition by showing alteration of root response upon external root exudate application in indirect GxG interactions (Biedrzycki et al., 2010; Semchenko et al., 2014). Furthermore, the composition of root exudates, which is genotype specific, may define the assembly of distinct soil microbial communities in the rhizosphere. Consequently, different genotypes in one population can be associated with different microbiota. Therefore, similar to soil microbiota mediated plant soil feedback (PSF) that has been abundantly documented at interspecific level and recognized in driving ecosystem processes (Klironomos, 2002; Kardol et al., 2006, 2007; Reinhart and Callaway, 2006; Petermann et al., 2008), soil microbiota may have a central role in mediating GxG interactions in natural plant communities. In our survey, four experiments reported indirect interactions for which the effect of soil microbiota conditioning by one genotype is tested on the growth response of another genotype (Bukowski and Petermann, 2014; Wagg et al., 2015; Bukowski et al., 2018; Burghardt et al., 2019). One of these studies suggested a role in maintaining genotypes coexistence (Bukowski et al., 2018), which is in line with recent theoretical demonstration of the putative role of microbes in altruism between organisms (Lewin-Epstein et al., 2017). Yet, the genuine role of PSF in shaping evolution of plant populations is largely unexplored (Wagg et al., 2015), and investigation of the role of microbiota on GxG interactions represents a future challenge.

Secondly, indirect interactions between genotypes can also be mediated by volatile organic compounds (VOCs) produced by plants both aboveground and underground. VOCs are known to act in neighbor detection (Ninkovic et al., 2019) and to affect intraspecific competition in crops (Ninkovic, 2003) and trees (Ormeño et al., 2007). In our survey, only three experiments focused on interactions through aerial VOCs (Karban et al., 2013 in *Artemisia tridentata*; Kalske et al., 2019 in *Solidago altissima;* and Shimola and Bidart, 2019 in *A. thaliana*). The cocktail of VOCs has been found to be highly variable among individuals and has been proposed to help distinguishing kin from stranger genotypes (Karban et al., 2014). Still, there is a caveat in understanding the exact role of VOCs in indirect GxG interactions in wild species, and how they may affect intraspecific communication between genotypes under natural conditions.

## Both Kin Cooperation and Overyielding Are Supported by Experiments

**FIGURE 4:**
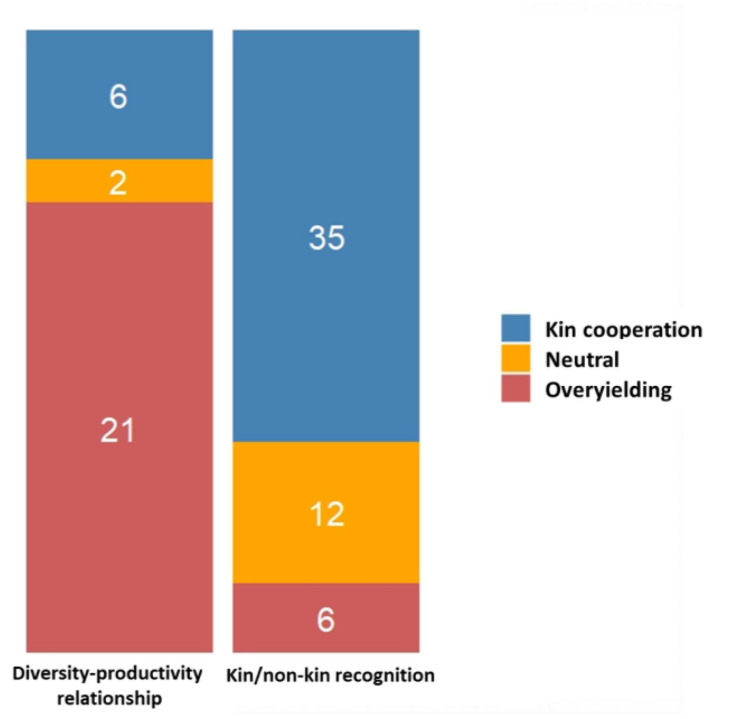
Stacked barplots illustrating the relative proportion of kin cooperation (KC), overyielding (OY) and neutral interactions between the ‘diversity-productivity relationship’ and ‘kin/non-kin recognition’ rationales. The number of studies supporting each type of interaction are indicated within each stack.

We divided the major conclusions from the 81 experiments underlying the two major rationales (kin/non-kin recognition *vs.* diversity-productivity relationship) into (i) *kin cooperation* (KC) for studies demonstrating positive interactions between kin genotypes, (ii) *overyielding* (OY) for studies where the mixture of genotypes was confirmed to be more productive than monocultures, and (iii) *neutral* for studies where no significant differences were observed between the treatments specified in the experiments. Among this set of 81 experiments, only ∼17% found no significant differences between treatments, while ∼50% reported KC and ∼33% studies reported OY (Supplementary Table 1). The relative proportion of KC, OY and neutral interactions in the survey can be matched to the two main rationales tested in the experiments. Experiments testing for kin/non-kin recognition confirmed more KC, while experiments aiming at testing diversity-productivity relationships, corroborated more cases of OY (contingency chi-squared test, χ^2^ = 31.68, df = 2, *P* = 1.32 E-07; Figure 4). However, we need to stress that the number of studies reporting no differences could be more than what is listed in this survey, owing to the high tendency of not publishing non-significant results.

Species having low dispersal rate have been theoretically proposed to allow for evolution of kin selection mechanisms as interacting individuals are likely to be relatives (Hamilton, 1964). Moreover, species having strong spatial population structure have been poised to have positive frequency dependent interactions among genetically related individuals (Ehlers and Bilde, 2019). However, in our survey, when considering all the experiments, the relative proportion of KC, OY or neutral interactions was not found dependent on the mode of seed dispersal (autochory *vs.* allochory, contingency chi-squared test, χ^2^ = 2.03, df = 2, *P* = 0.362; Supplementary Figure 1) or on the geographical scale of sampling (one population *vs.* multiple populations, contingency chi-squared test, χ^2^ = 3.34, df = 2, *P* = 0.188; Supplementary Figure 2). Removing *A. thaliana* from the list yielded similar results for these proportions (autochory *vs.* allochory, contingency chi-squared test, χ^2^ = 0.67, df = 2, *P* = 0.714; one population *vs.* multiple populations, contingency chi-squared test, χ^2^ = 1.08, df = 2, *P* = 0.582; Supplementary Figures 1, 2).

**FIGURE 5:**
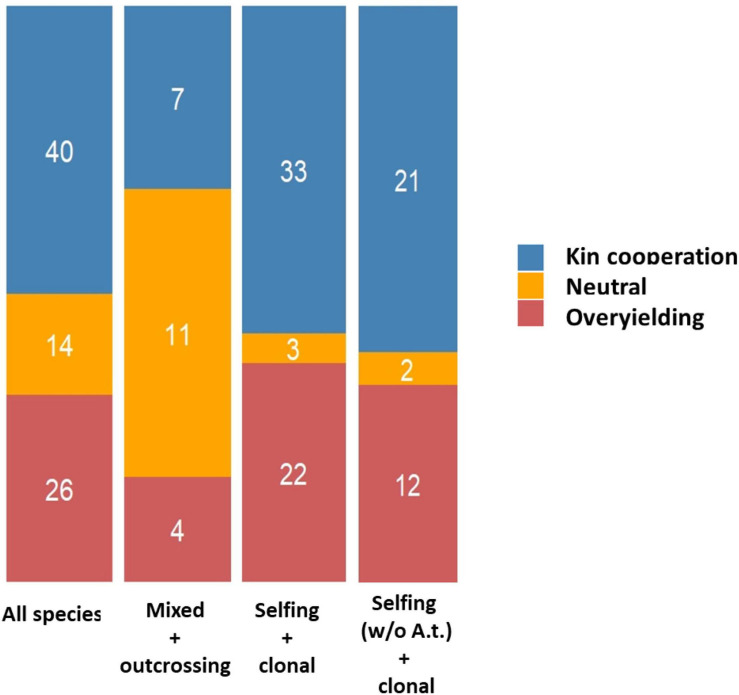
Stacked barplots illustrating the different outcomes of GxG according to the breeding system of species studied. MIXED + OUTCROSSING: species with an outcrossing breeding system or with a mixed breeding system. SELFING + CLONAL: species with a selfing breeding system or a vegetative reproduction. SELFING W/O A.T. + CLONAL: species with a selfing breeding system (without considering *Arabidopsis thaliana*) or a vegetative reproduction.

In species with a high selfing rate, the presence of neighbors of high genetic relatedness in the vicinity of seed-dispersing mother plants is expected in natural populations, which may allow for the prevalence of kin selection mechanisms for increasing one’s inclusive fitness (Dudley et al., 2013; Ehlers and Bilde, 2019). The relative proportions between the three types of interactions (KC, OY or neutral) were significantly dependent on the breeding system of the plant species studied (selfing and clonal *vs.* outcrossing and mixed, χ^2^ = 22.23, *P* = 1.48 E-05). KC was detected in 56.9 and 31.8% of the experiments conducted on species with a selfing/clonal and outcrossing/mixed breeding system, respectively (Figure 5). This effect remained significant even upon the removal of *A. thaliana* from the list (χ^2^ = 15.04, *P* = 5.39 E-04).

**FIGURE 6:**
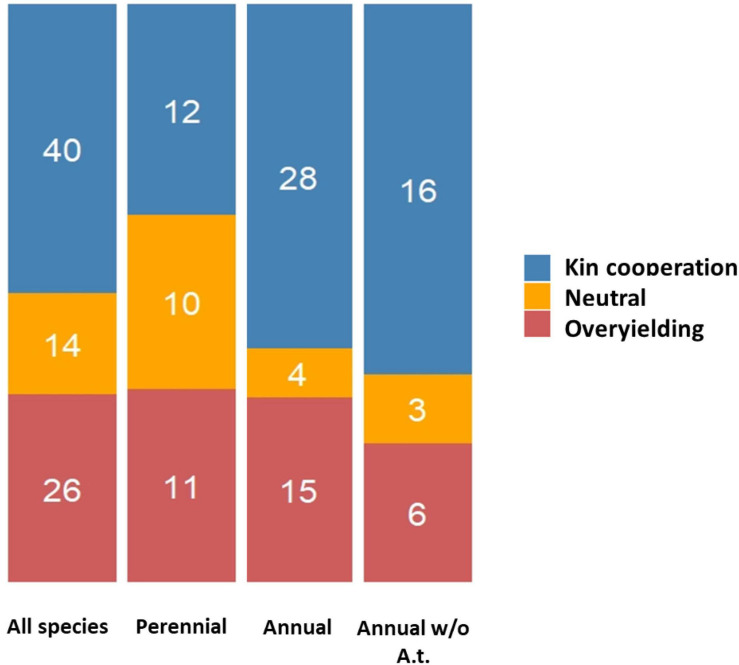
Stacked barplots illustrating the different outcomes of GxG interactions according to the annual *vs.* perennial strategy of species studied (with and without including *Arabidopsis thaliana*). The number of studies supporting each type of interaction are indicated within each stack.

Species having an annual life cycle are known to be commonly composed of genetically related individuals, especially in cases of limited seed dispersal and self- fertilization (Cheplick and Kane, 2004). Therefore, kin selection mechanisms may prevail for annual plants. In our survey, the relative proportions between the three types of interactions (KC, OY or neutral) were found to be dependent on the annual *vs.* perennial life history strategy of the plant species considered (χ^2^ = 7.36, *P* = 0.025), with KC detected in 59.7 and 36.4% of the experiments conducted on annual and perennial species, respectively (Figure 6). This difference between these two life history strategies was however not significant upon removal of *A. thaliana* from the list of species considered (χ^2^ = 4.80, *P* = 0.091; Figure 6).

At the interspecific level, increase in magnitude of positive interactions has been linked to varying levels of abiotic (Maestre et al., 2009) and biotic stresses (Smit et al., 2009) in various studies. In our survey, we identified two studies reporting a shift from KC to OY occurring between the same genotypes in response to a particular environmental factor. Firstly, in *A. thaliana*, genotypes performed better with kin under ambient CO_2_ concentrations, while genotypic mixtures were favored under elevated CO_2_ concentrations (Andalo et al., 2001). Secondly, in *Phalaris arundinacea*, genotypes performed better with kin in disturbed plots (with all native vegetation removed), while genotypic mixtures were confirmed to perform better in undisturbed plots (Collins et al., 2018). Both experiments provide evidence for the existence of similar dynamics of intraspecific positive interactions among different species in varying environments.

## Future Avenues

**FIGURE 7:**
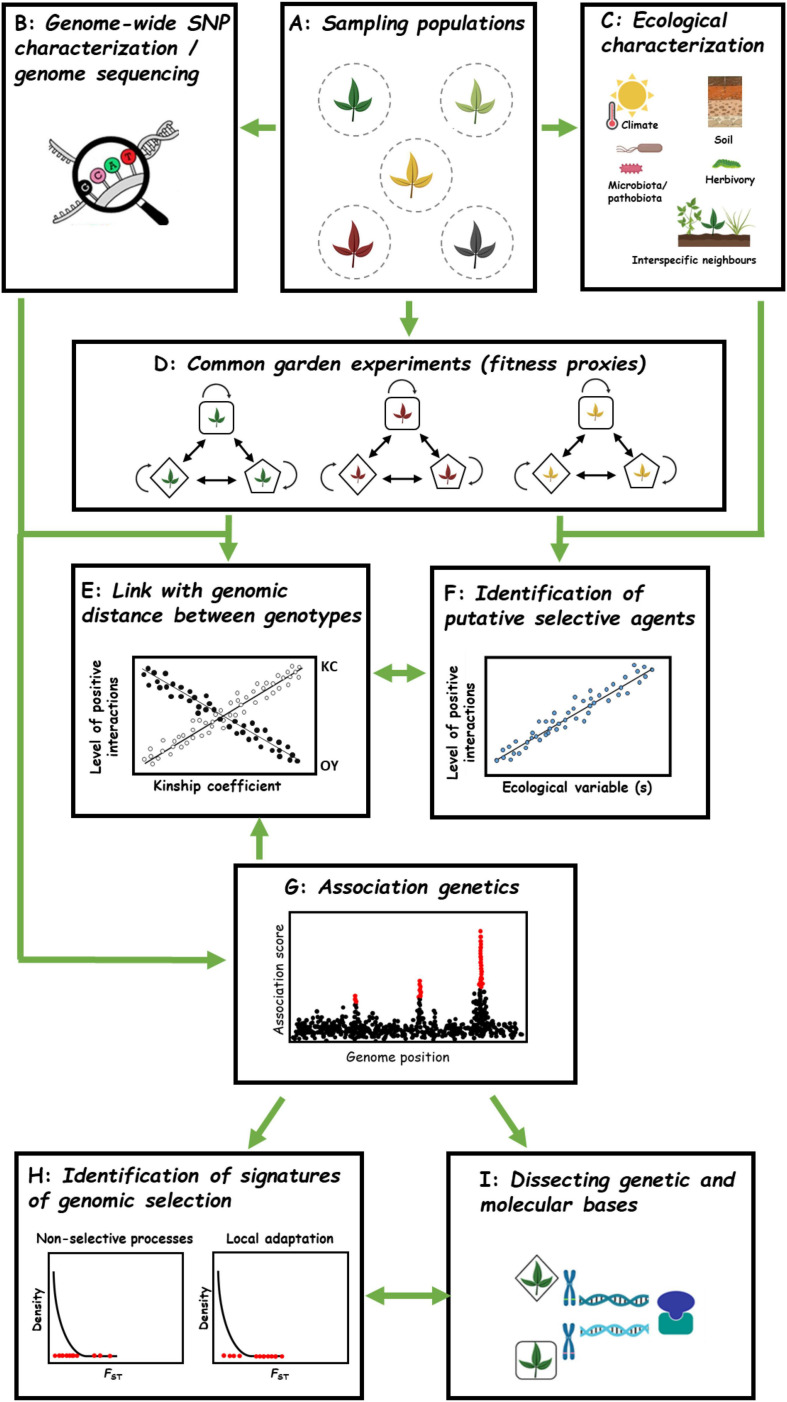
Future avenues for studying GxG interactions among wild plant populations. **(A)** Collecting multiple genotypes from several natural populations. **(B)** Genome sequencing/Genome-wide SNP characterization of all genotypes coming from different populations. **(C)** Characterizing abiotic and biotic ecological factors. **(D)** Conducting common garden experiments to dissect fitness outcomes in pairwise interactions among genotypes from one population (intragenotype and intra-population intergenotype combinations). Different genotypes are represented by different shapes. **(E)** Linking genomic distance with levels of positive interactions exhibited in pairwise GxG interactions. **(F)** Identifying putative ecological driver(s) associated with among-population variation of positive interactions. **(G)** Genome Wide Association mapping to identify genomic regions underlying positive interactions. **(H)** Enrichment analysis in signatures of selection, e.g., spatial genetic differentiation estimated by *F*_ST_. **(I)** Cloning and functional characterization of genes underlying QTLs associated with positive interactions.

Despite the demonstration of positive interactions in 36 species (Supplementary Table 1), the underlying adaptive genetic architecture is still an open question. In our survey, this challenge was addressed in only one study, with the identification of a single major QTL underlying kin cooperation in *A. thaliana* (Wuest and Niklaus, 2018). While informative, the low number of RILs used (*N* = 37) precluded a proper characterization of the genetic architecture (Keurentjes et al., 2007). In addition, remaining questions include (i) testing whether polymorphic genes involved in positive interactions have been shaped by natural selection and (ii) identifying the ecological factors driving adaptive KC or OY (Subrahmaniam et al., 2018; Frachon et al., 2019). Here, we therefore introduce a framework in order to establish a genomic map of local adaptive positive plant–plant interactions (Figure 7).

The first step would be collecting genetic material from multiple local populations (Figure 7A). Then, the genomic characterization of the plant material could be conducted either by genome-wide SNP characterization or whole genome sequencing methodologies. This will not only allow estimating the genetic relatedness among genotypes within and between local populations but will also be essential to run GWA mapping (see below, Figure 7B). Simultaneously, an extensive *in situ* characterization of both abiotic (e.g., climate, soil physico-chemical properties) and biotic (e.g., plant communities, herbivore communities, as well as microbiota including commensal, pathogenic and symbiotic microbes) factors should be conducted for each population (Figure 7C). While abiotic stress characterization is facilitated by publicly available databases for climate and dedicated platforms for soil physico-chemical properties, characterizing biotic factors can still be strenuous and time consuming. However, with the ever-decreasing cost of next-generation sequencing (NGS) technologies, describing the taxonomic and functional networks (microbes, plants, insects…) of hundreds of wild plant populations is not out of reach (Bartoli et al., 2018; Frachon et al., 2019). While collecting plant material and accumulating genomic, phenotypic and ecological data for tens to hundreds of populations represents a monumental task, this has already been achieved for a few wild plants including *A. thaliana* (Fournier-Level et al., 2011; Hancock et al., 2011; Brachi et al., 2013; Bartoli et al., 2018; Frachon et al., 2018, 2019) and sunflower (Todesco et al., 2020).

In order to estimate the natural variation of GxG interaction patterns among populations, the next step would be to conduct common garden experiments by estimating differences at the intra-population level between intra-genotypic interactions (monoculture) and inter-genotype pairwise interactions (Figure 7D). Although the best strategy would be to perform experiments in the native habitats from which the populations have been collected, this approach may not be manageable for a larger number of populations.

Kin recognition and elbow-room hypotheses make opposite predictions about the relationship between genetic distance and the extent of positive interactions between two genotypes (Figure 7E). Having genome sequence information of genotypes, linking level of positive interactions to the genomic distance of interacting genotypes could help validating either hypothesis for explaining positive interactions between populations (Figure 7E). Additionally, identification of ecological factors associated with positive interactions between populations could be of interest in understanding how positive interactions (KC or OY) may evolve under different environmental selection pressures in natural populations (Figure 7F) and designing experiments to test the relevance of the stress gradient hypothesis at the intraspecific level.

As a next step, a GWA mapping approach could be adopted to fine map genomic regions associated with natural variation in the level of positive interactions among populations (Figure 7G). GWA mapping has previously proven to be a powerful approach to identify QTLs associated with plant–plant interactions at the intraspecific and interspecific levels in many crop species (Subrahmaniam et al., 2018) as well as in *A. thaliana* (Baron et al., 2015; Frachon et al., 2017, 2019). The detected QTLs can in turn help to reliably discriminate between the three main hypotheses about the genetic architecture underlying positive interactions within species, that is (i) kin selection wherein a large chunk of the genome is shared among cooperating partners (polygenic architecture; Hamilton, 1964), (ii) Green beard effect with cooperative genotypes sharing the same allele at one or few QTLs identified (Dawkins, 1976), and (iii) complementarity where cooperative genotypes have different alleles at single or few QTLs identified (D’Souza et al., 2018).

Such QTLs can then allow identifying signatures of selection at the genomic level (Figure 7H). For instance, the type and strength of selection can be addressed by testing for any overlaps and enrichments between QTLs underlying positive interactions and genomic regions showing signatures of long-term (e.g., hard sweeps) or short-term selection (e.g., local adaptation with *F*_ST_) (Fournier-Level et al., 2011; Hancock et al., 2011; Horton et al., 2012; Brachi et al., 2015; Frachon et al., 2017; Figure 7H).

After confirming the adaptive status of the genetic basis associated with natural variation of positive interactions, one could take the final steps toward understanding the genetic and molecular mechanisms (Figure 7I). For this, QTL cloning can be achieved by characterization of mutants (T-DNA, EMS…) or generation of transgenic lines with gain or loss of functions of the gene(s) of interest.

Altogether, combining ecology and evolutionary biology, along with quantitative genetics, genomics and molecular biology represents an unprecedented opportunity to establish the genetic and molecular picture of positive GxG interactions in wild plant species in their natural ecological landscape.

## Author Contributions

All authors listed have made a substantial, direct and intellectual contribution to the work, and approved it for publication.

## Conflict of Interest

The authors declare that the research was conducted in the absence of any commercial or financial relationships that could be construed as a potential conflict of interest.

## Footnotes

1 https://www.researcher-app.com/

## Supplementary Material

Supplementary Table 1 Survey of GxG interaction studies in herbaceous wild plant species. The first six columns (columns A–F) indicate the serial number, the list of different plant species studied, their botanical family, main breeding system, life cycle and natural seed dispersal mode. Next six columns (columns G–L) describe the genetic material used including the main type of genetic variation studied (here experimental variation indicates use of F_2_ population, Recombinant Inbred Lines or Introgression Lines, while artificial variation includes use of mutant lines), number of genetic lines sampled (here NA indicates not available), number of populations sampled (here NA indicates not available and NR indicates not relevant), the scale of sampling (here WW indicates worldwide sampling), whether genetic differences between the lines were tested or assumed, and the habitats from which these lines were sampled from. Following 15 columns (columns M–AA) are divided to describe the experimental design of the studies including the main rationale, whether the GxG interactions were tested directly or indirectly, the experimental design (here *solo* refers to treatments where genotypes were grown alone, *intra* refers to ‘intragenotype’ treatment where two individuals of the same genotype were placed together and *inter* refers to ‘intergenotype’ treatment where two different genotypes were placed together; in the ‘inter’ column, ‘x’ in red indicates a mixed polyculture treatment) and conditions under which the experiments were performed (here GH indicates greenhouse and GC indicates growth chamber). Additionally, details about the specific treatments applied and the traits measured are listed. Next three columns (columns AB–AD) detail the main ecological questions addressed in each study, major findings with respect to intraspecific plant–plant interactions and the main type of positive interaction observed in each experimental study. Here KC indicates kin cooperation, OY indicates overyielding, coop indicates studies where positive interactions were identified. However, due to lack of genetic information, these latter studies could not be categorized in either KC or OY. The last three columns (columns AE–AG) list the reference name, year of publication, and the journal of publication (Tonsor, 1989; Mahall and Callaway, 1991; Burt and Bell, 1992; Thomas and Bazzaz, 1993; Mahall and Callaway, 1996; Ronsheim and Anderson, 2001; Rautiainen et al., 2004; Falik et al., 2006; Dudley and File, 2007; Semchenko et al., 2007; Lankinen, 2008; Murphy and Dudley, 2009; Aguilera et al., 2011; Bhatt et al., 2011; Biernaskie, 2011; Markham and Halwas, 2011; Drummond and Vellend, 2012; Milla et al., 2012; Creissen et al., 2013; Tomimatsu et al., 2014; Yang et al., 2015; Corliss and Sultan, 2016; Huber et al., 2016; Palmer et al., 2016; Abd El-Gawad et al., 2017; Jie et al., 2017; Li and Xu, 2018; Torices et al., 2018; Baker et al., 2019; Smith et al., 2019; Tomiolo et al., 2019; Zhang et al., 2019; Goddard et al., 2020; Yamawo and Mukai, 2020).

Supplementary Table 2 List of different traits measured in GxG interaction studies listed in the literature survey. The serial number and list of species studied in the 91 experiments are listed in the first two columns (columns A and B). The next four columns (columns C–F) describe the measured trait categories that have been divided into root, shoot, life history and seed production related traits. Following this, the columns (columns G–J) summarize the total number of traits studied in each trait category within the experiments. The next two columns detail the extended phenotypes, if recorded within the experiment (columns K and L). Following this, the total number of traits recorded within the experiment are listed (column M). Lastly, the two columns (columns N and O) list the reference name, and the year of publication.
