# Supplementary material for: Toward Unifying Evolutionary Ecology and Genomics to Understand Positive Plant–Plant Interactions Within Wild Species
Source: Front Plant Sci. 2021 Jul 9;12:683373. doi: 10.3389/fpls.2021.683373 (PMC8299075; doi:10.3389/fpls.2021.683373)

**Towards unifying evolutionary ecology and genomics to understand positive plant-plant interactions within wild species**

Harihar Jaishree Subrahmaniam^1*^, Dominique Roby^1^ and Fabrice Roux^1*^

^1^LIPME, Université de Toulouse, INRAE, CNRS, Castanet-Tolosan, France

* To whom correspondence should be addressed. E-mail: [jaishree.subrahmaniam@gmail.com](mailto:jaishree.subrahmaniam@gmail.com), fabrice.roux@inrae.fr

**Supplementary Figure 1.** Stacked barplots illustrating the different outcomes of GxG based on the mode of seed dispersal of the studied species (with and without *Arabidopsis thaliana*). The number of studies found supporting each type of interaction are indicated within each stack.


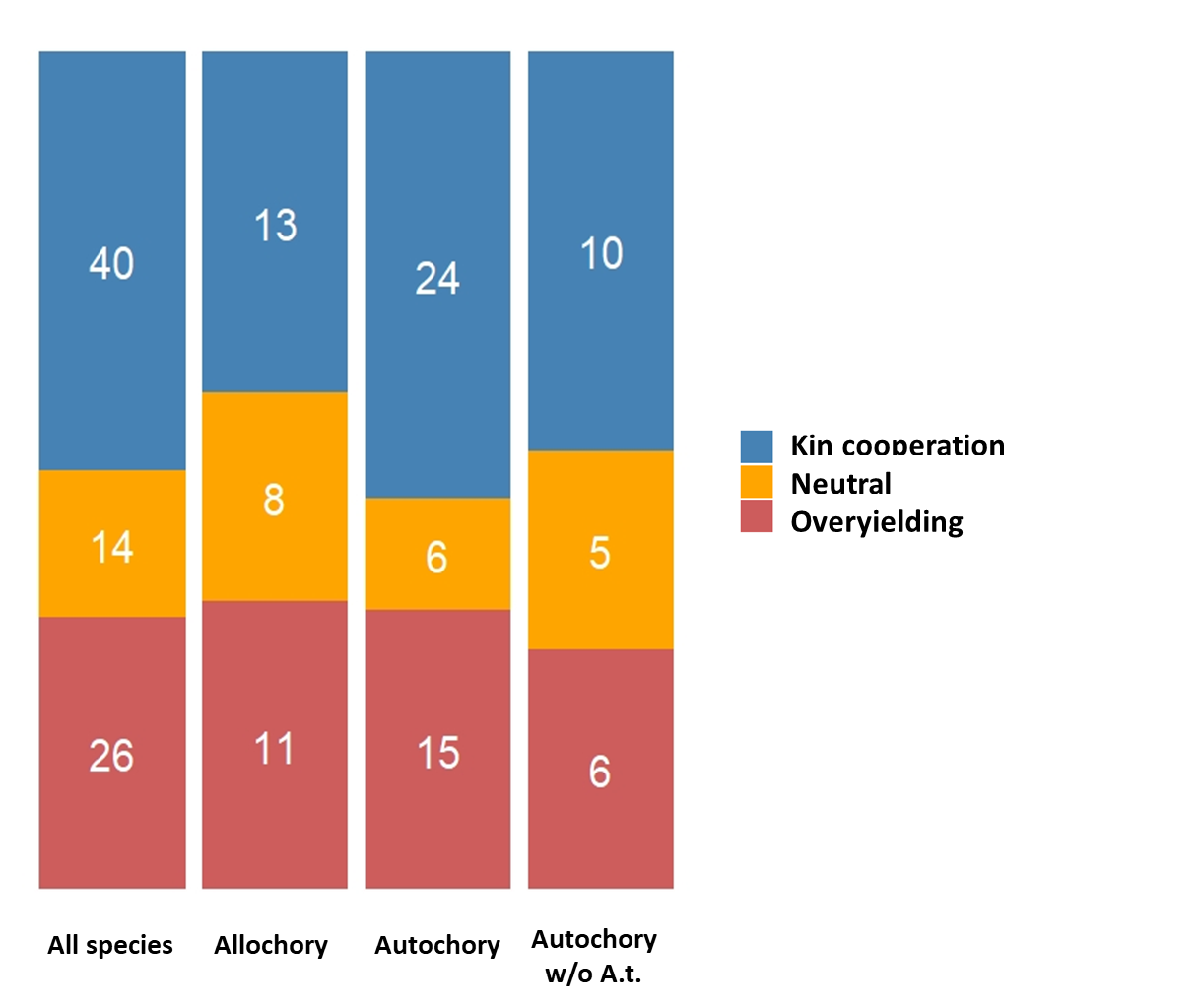


**Supplementary Figure 2.** Stacked barplots illustrating the different outcomes of GxG based on the geographic scale of sampling (genotypes collected from one population *vs.* multiple populations: with and without *Arabidopsis thaliana*). The number of studies found supporting each type of interaction are indicated within each stack.


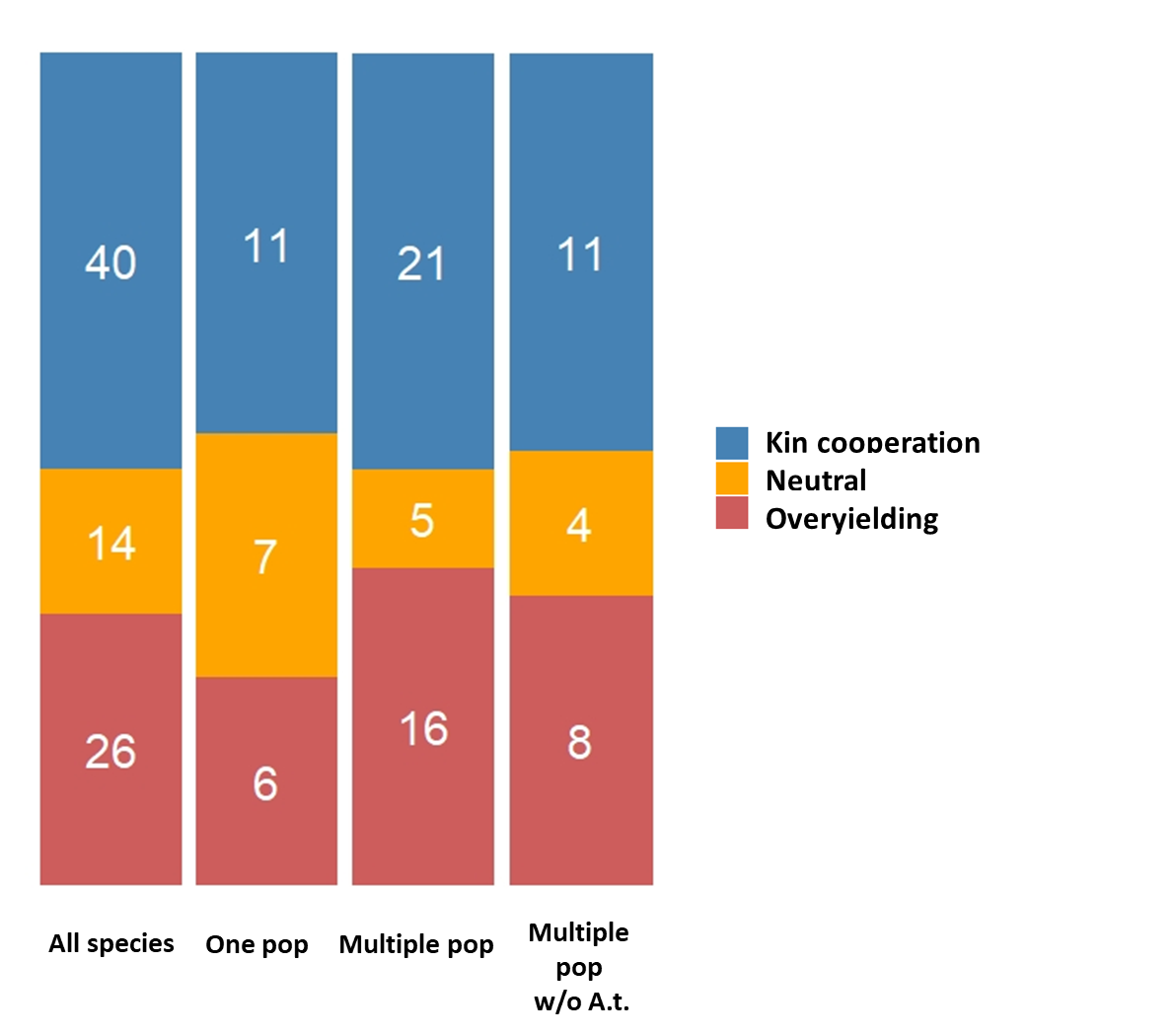

Supplement: Supplementary file 2 [file Data_Sheet_2.docx]
